# Stop Auditing Mothers and Start Auditing Systems

**DOI:** 10.1111/mcn.70267

**Published:** 2026-10-15

**Authors:** Jimmy Chun Yu Louie

**Affiliations:** ^1^ Department of Biomedical, Health and Exercise Sciences, School of Health Sciences Swinburne University of Technology Hawthorn Victoria Australia

**Keywords:** breastfeeding, equity, health policy, health systems, infant feeding, lactation support, maternal and child nutrition

## Abstract

National breastfeeding statistics are conventionally read as a measure of maternal behaviour, and the dominant policy response has been to educate and encourage individual women. This Perspective argues that breastfeeding statistics should be interpreted primarily as indicators of system performance rather than maternal behaviour. In high‐income countries, much early cessation occurs among women who intended to continue breastfeeding but could not obtain timely, effective support. The resulting shortfall therefore reflects constrained choice as much as personal preference. That both the systems framing and the intention‐to‐cessation indicator already exist in the literature is acknowledged directly: surveillance research has measured the gap, described its risk factors, and mapped its social patterning. Viewed through a quality‐and‐safety lens, unwanted early cessation has many of the characteristics of a preventable harm. It is common, patterned by identifiable failures of care, and partly avertable with known interventions such as evidence‐based maternity practices, proactive post‐discharge contact, adequate paid leave, and protection from substitute marketing. The contribution of this Perspective is not a new metric, but a new use for an existing one. I argue that unwanted early cessation should be treated as an accountable quality indicator within the quality‐and‐safety systems already used for other preventable harms. Rather than remaining a descriptive statistic, it should become a measure for which health services, employers, and policymakers share accountability. The paper also addresses how intention should be measured, considers the risks of increased surveillance, safeguards maternal autonomy, avoids shifting blame onto clinicians, and distinguishes challenges in high‐income settings from priorities in lower‐income contexts.

## Key Messages

- Much recorded early breastfeeding cessation occurs among women who intended to continue breastfeeding but could not obtain the support needed to do so.
- The intention‐to‐cessation gap is already measured, but no institution is accountable for it.
- Unwanted early cessation can be managed as a preventable harm, using the same accountability and quality‐improvement tools applied elsewhere in healthcare.

Few early‐life practices are monitored as closely as breastfeeding. The World Health Organization recommends exclusive breastfeeding to 6 months and continued breastfeeding to 2 years and beyond (World Health Organization 2003), and national surveys track initiation, exclusivity at 6 months, and any breastfeeding at 1 and 2 years. The 2016 Lancet Breastfeeding Series quantified what the shortfall costs, estimating that scaling breastfeeding towards universal levels could avert roughly 823,000 child deaths annually, alongside substantial cognitive and economic losses (Rollins et al. 2016; Victora et al. 2016). The 2023 Series revisited these questions and shifted the emphasis decisively towards commercial and structural determinants, describing infant feeding as increasingly challenged in a market‐driven world (Baker et al. 2023; Pérez‐Escamilla et al. 2023). Progress since has been genuine but slow, and most countries still sit below target (UNICEF and World Health Organization 2023).

Yet breastfeeding statistics are usually interpreted as measures of maternal behaviour. When rates fall short of targets, the response is often more education, more counselling, and more encouragement directed at mothers themselves (McFadden et al. 2019; Rollins et al. 2016). The logic is intuitive. If women knew more, or were encouraged more warmly, more of them would breastfeed for longer. Yet breastfeeding rates remain below recommended levels in most countries despite decades of education and promotion efforts (Rollins et al. 2016; Victora et al. 2016), which invites a less comfortable question about the unit we have chosen to measure. *When a national statistical office reports that most mothers stop breastfeeding before the recommended point, whose performance is that number actually describing?*

## 1 What Is Already Known, and What This Perspective Adds

Breastfeeding outcomes are widely recognised as products of the systems surrounding families rather than maternal determination alone. A decade ago, an editorial in this journal argued that breastfeeding programmes operate in a complex adaptive world and that scaling them demands systems thinking and well‐validated participatory decision tools rather than exhortation (Pérez‐Escamilla and Hall Moran 2016). The 2023 Lancet Breastfeeding Series went considerably further, documenting how corporate power, weak regulation, and structural barriers shape infant feeding, and setting out what confronting them would require (Baker et al. 2023; Pérez‐Escamilla et al. 2023). A recent National Academies review reached parallel conclusions for the United States, framing the task as supporting families rather than instructing mothers (National Academies of Sciences, E., Medicine, Division of, B., Social, S., Education, Board on Behavioral, C.,… Support Across the United States: An, A 2025).

The intention‐to‐cessation gap is also well established. Analyses of the Infant Feeding Practices Study II by investigators at the Centers for Disease Control and Prevention found that among women who intended to breastfeed exclusively, only 32.4% achieved their intended duration, and that receiving supplemental feeds in hospital was the practice most strongly associated with failing to meet that intention (Perrine et al. 2012). Work published in this journal subsequently showed that meeting intentions differs by race and ethnicity even where the intentions themselves do not, with non‐Hispanic Black and Hispanic women significantly less likely to meet goals they had stated at the same rate as others (Hamner et al. 2021). Later analyses of low‐income women linked specific maternity care practices to whether prenatal intentions were met (Beauregard et al. 2022). The indicator, its risk factors, and its social patterning are therefore established rather than proposed here.

What remains missing is accountability for the gap. The intention‐to‐cessation gap is routinely measured in surveillance systems and research cohorts, but it is not tied to any accountable institution. The argument advanced here is that it should change status: unwanted early cessation should be treated as a reportable quality indicator within the quality‐and‐safety systems already used for other preventable harms, with clear accountability, facility‐level and employer‐level reporting, and a place in maternity accreditation. The distinction is between knowing that most women do not meet their goals and holding particular institutions answerable for that shortfall. Measurement alone does not create accountability, and that step has yet to be taken.

## 2 What the Surveys Are Really Recording

Consider what sits underneath the headline duration figure. In high‐income countries with reliable data, a large majority of women initiate breastfeeding (Victora et al. 2016), and a substantial proportion stop earlier than they themselves had planned, with around 60% of women in one national cohort ceasing before their desired duration (Odom et al. 2013). Studies from several countries converge on the same pattern. Mothers cite pain, cracked nipples, perceived insufficient milk, worry about infant weight, exhaustion, return to work, and unhelpful or contradictory professional advice as reasons for stopping (Brown et al. 2016; Newby and Davies 2016; Odom et al. 2013), and many report doing so with regret or having stopped before their desired duration (Odom et al. 2013). The more informative measure is the gap between intended and achieved duration. This gap reflects not realised preference but constrained choice (Odom et al. 2013; Perrine et al. 2012).

This reframing changes the meaning of the metric. Duration statistics tell us what happened. Measured against women's own goals, they also tell us where support failed (Brown et al. 2014; Odom et al. 2013). The two readings point policy in opposite directions. The first invites us to persuade women to want breastfeeding more. The second obliges us to ask why so many who already wanted it could not sustain it (Gianni et al. 2019; Odom et al. 2013). Much of the shortfall we record is better described as constrained choice (Hausman 2012), and constraint is something systems impose rather than something individuals possess.

None of this argues that every woman intends to breastfeed, or should. Some women choose formula freely and for good reasons, and that choice deserves respect and unqualified support (World Health Organization WHO, and UNICEF 2018). The point is narrower and more troubling. A sizeable share of recorded early cessation occurs among women who wanted the opposite outcome and could not obtain the help that would have delivered it (McFadden et al. 2019; Odom et al. 2013). By focusing only on the endpoint, measurement can recast system failures as individual ones (Pérez‐Escamilla et al. 2016; Rollins et al. 2016).

## 3 A Different Unit of Analysis

The determinants of whether a woman initiates, exclusively breastfeeds, and continues have been mapped in detail, and most lie outside the individual mother. Hospital birth practices, early skin‐to‐skin contact, rooming‐in, the clinical governance of supplementation, timely access to skilled lactation support, paid maternity leave of adequate length and income replacement, workplace flexibility, protection from aggressive substitute marketing, partner and community support, and the mother's own physical and mental health all shape the outcome (Pérez‐Escamilla et al. 2012; Rollins et al. 2016; Sinha et al. 2015). The 2016 Series authors named this the enabling environment and argued for investment across it (Rollins et al. 2016), and the 2023 Series subsequently sharpened the commercial and regulatory components of that environment (Baker et al. 2023). The next step is to align accountability with the factors that shape outcomes. If breastfeeding outcomes are shaped largely by clinical, workplace, commercial, and policy environments, they should be used to assess those environments rather than the women navigating them.

Figure 1 sets out this logic as a systems model, with the mother‐infant dyad embedded in interacting clinical, workplace, community, commercial, and policy layers. The importance of the model lies not in its novelty but in its implications for accountability. If breastfeeding is a systems output, then a low national figure is diagnostic information about the system, in the same way that a rising rate of surgical site infection is diagnostic information about a surgical service rather than a character flaw in patients (Reason 2000; Rollins et al. 2016).

**Figure 1:**
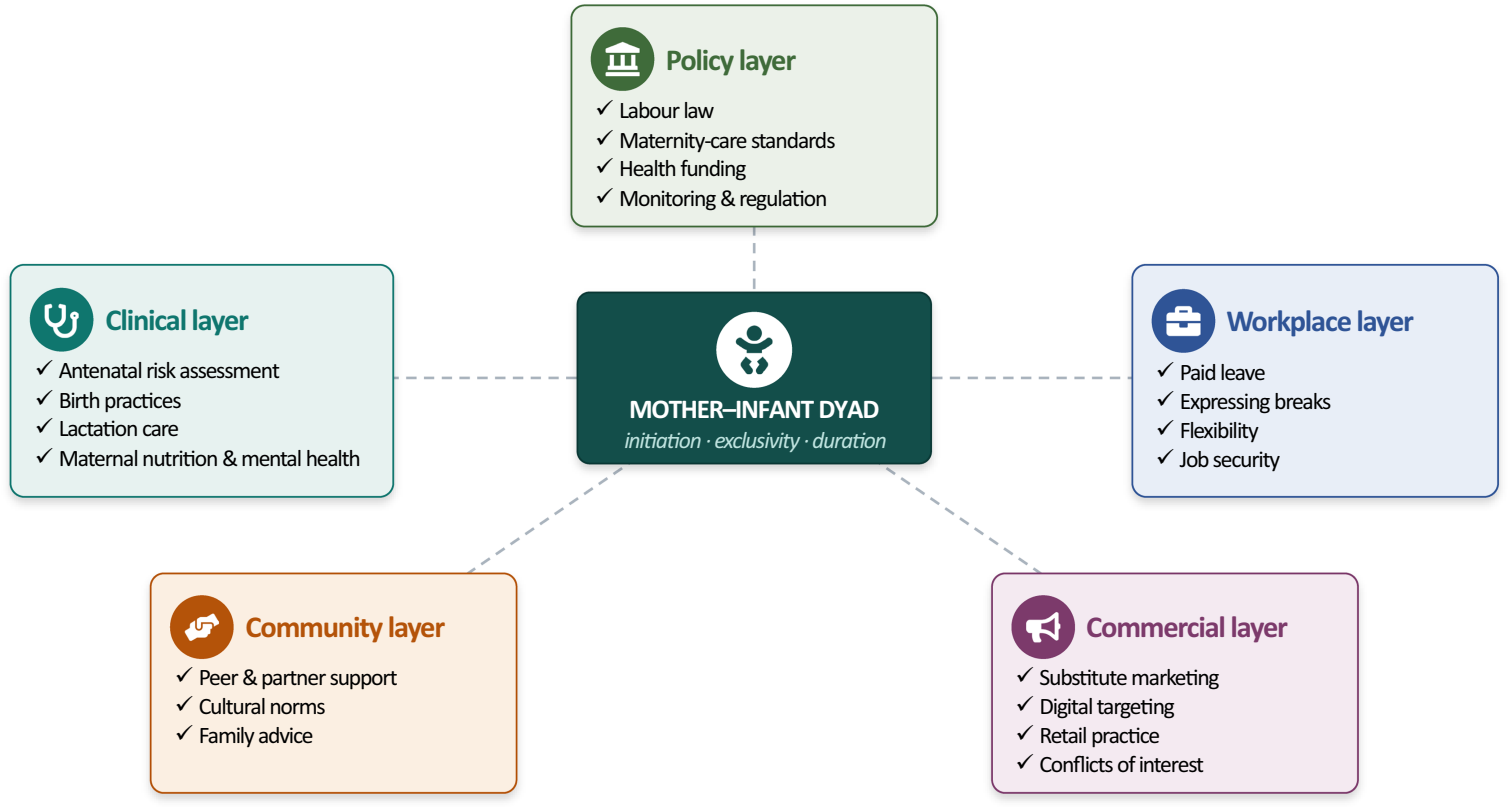
Breastfeeding as a systems output. The mother–infant dyad sits within five interacting layers, each of which shapes whether breastfeeding is initiated, exclusive, and sustained: a clinical layer, a workplace layer, a community layer, a commercial layer, and a policy layer. Education remains useful but is insufficient when the surrounding layers make breastfeeding difficult to initiate or maintain.

Evidence supporting these interventions is substantial. Baby‐friendly hospital practices raise initiation and duration, particularly when delivered as an integrated bundle rather than a checklist of isolated steps (Pérez‐Escamilla et al. 2016; Sinha et al. 2015; World Health Organization, and UNICEF 2009). Paid maternity leave is associated with better breastfeeding outcomes (Chai et al. 2018; Huang and Yang 2015) and, in quasi‐experimental work, with lower infant mortality, with stronger effects where leave is longer and better paid (Nandi et al. 2016). However, implementation remains the principal challenge. A hospital can hold baby‐friendly designation and still discharge a woman at 24 h with a poor latch, an unsettled infant, and a leaflet. A workplace can advertise a lactation policy while offering a locked storeroom and a pumping break contingent on a sympathetic supervisor. Designation and policy are inputs. What remains poorly measured is whether mothers receive effective support when they need it.

### 3.1 Treating Unwanted Cessation as a Preventable Harm

The evidence points to a straightforward question. Few areas of healthcare respond to a persistent gap between recommended and achieved care primarily through patient education alone. In cancer care, cardiology, or surgery, a systematic gap between recommended and achieved care triggers audit, root‐cause analysis, and a search for the structural reasons patients are not receiving what works (Pronovost 2003; Reason 2000). Breastfeeding remains an important exception, and the exception is sustained by the habit of classifying the outcome as behaviour rather than care (Pérez‐Escamilla et al. 2012; Rollins et al. 2016).

Should unwanted early cessation be treated like other preventable harms in healthcare? Unwanted early cessation meets many of the criteria used to define preventable harm. It is common (Odom et al. 2013; Perrine et al. 2012), measurable (World Health Organization WHO, and UNICEF 2018), socially patterned (Gianni et al. 2019; McFadden et al. 2019), linked to identifiable failures of support, and partly avoidable through known interventions, including early skin‐to‐skin contact, avoidance of clinically unnecessary supplementation, skilled latch assessment, proactive postnatal contact, and peer support (Pérez‐Escamilla et al. 2016; Shakya et al. 2017; Sinha et al. 2015). Health systems already have established methods for addressing events of this kind. They count such occurrences without blaming the individual affected, trace them to their systemic causes, and hold institutions accountable for reducing them (Pronovost 2003; Reason 2000). Applying that machinery to infant feeding would not moralise about mothers. Instead, it would shift accountability from mothers to the institutions that shape their opportunities to succeed.

The practical implications are straightforward but important. Health systems do not need a new bureaucracy or surveillance platform. They already audit preventable outcomes, investigate unwarranted variation, and use quality indicators to guide improvement. The proposal here is simply to place unwanted early cessation within that existing framework. A maternity service with a persistently high intention‐to‐cessation gap would be expected to review discharge practices, access to lactation support, supplementation policies, staffing, and continuity of care, just as it would for any other preventable adverse outcome. The aim is not punishment but improvement: making avoidable cessation visible, measurable, and actionable.

This shift in accountability is central to the argument. If breastfeeding outcomes are understood as system outputs, responsibility extends beyond mothers to the institutions that shape feeding environments, including hospitals, governments, employers, professional bodies, and data systems. The task is not to identify who should be blamed, but to identify which parts of the system can most effectively reduce avoidable cessation. Concretely, this means that facilities would report unwanted early cessation alongside other quality indicators, that unexplained variation would trigger review and improvement efforts, and that maternity accreditation would assess not only breastfeeding policies but also whether women are able to meet their feeding goals.

### 3.2 What to Measure Instead

Reframing the event changes what a serious monitoring system would collect. Current indicators tell us how many women breastfeed and for how long. They are silent on whether women received timely help, whether in‐hospital supplementation was clinically justified or merely convenient, whether parents were exposed to commercial influence at vulnerable moments, whether low‐wage workers had any real access to lactation breaks, and, most tellingly, whether women stopped earlier than they wished. Table 1 sets out the shift from a promotion model to a systems model across the main policy domains and pairs each with indicators that capture conditions rather than compliance.

**Table 1:** From breastfeeding promotion to breastfeeding‐systems redesign.

Policy domain	Dominant promotion model	Systems‐redesign approach	Indicators to monitor
Antenatal care	Provide information on benefits	Assess feeding intentions and risks; address maternal nutrition, mental health, and prior feeding experience	Documented feeding plan and intended duration, recorded as revisable referral for complex risk; access to dietetic and lactation care
Birth hospital	Encourage early breastfeeding	Embed evidence‐based maternity practices, skilled latch support, and clinically governed supplementation	Skin‐to‐skin and rooming‐in rates; proportion of supplementation clinically justified; staff competency
Post‐discharge care	Provide a helpline number	Offer proactive contact in the first week; rapid access for pain, mastitis, supply concerns, and faltering weight	Time from discharge to first contact; wait for acute lactation help; facility‐level unwanted early‐cessation rate
Workplace	Advise mothers to express milk	Mandate adequate paid leave, lactation breaks, private space, storage, and supervisor accountability	Paid‐leave coverage and adequacy; lactation‐room quality; break access; complaints and enforcement
Commercial environment	Warn parents about marketing	Enforce marketing restrictions across health services, retail, and digital platforms	Code violations; measured digital‐advertising exposure; conflict‐of‐interest disclosure
Equity	Universal messaging	Proportionate support for families facing the steepest barriers	Intention‐to‐cessation gap and duration disaggregated by income, ethnicity, migration status, age, geography
Clinical complexity	Generic advice	Tailored pathways for preterm birth, bariatric surgery, hypertensive disorders, diabetes, mental illness, and infant illness	Referral completion; donor‐milk access; specialist nutrition support

Three measures would make the biggest difference. First, the intention‐to‐cessation gap, measured as the proportion of women who stop before their own stated goal, should be elevated to a primary indicator and disaggregated by income, ethnicity, migration status, maternal age, and geography, a disaggregation that existing analyses show is far from cosmetic (Hamner et al. 2021). It is the closest thing we have to a direct readout of unmet support. Second, time‐to‐support metrics, such as the interval from discharge to the first proactive lactation contact and the wait for help with an acute problem, would expose the postnatal week that current indicators ignore. Third, facility‐level and employer‐level adherence to the practices known to matter would locate accountability precisely, replacing the binary of designation with a graded measure of what was actually delivered.

Figure 2 illustrates these indicators as a breastfeeding‐support cascade, identifying the predictable points at which women lose access to effective support.

**Figure 2:**
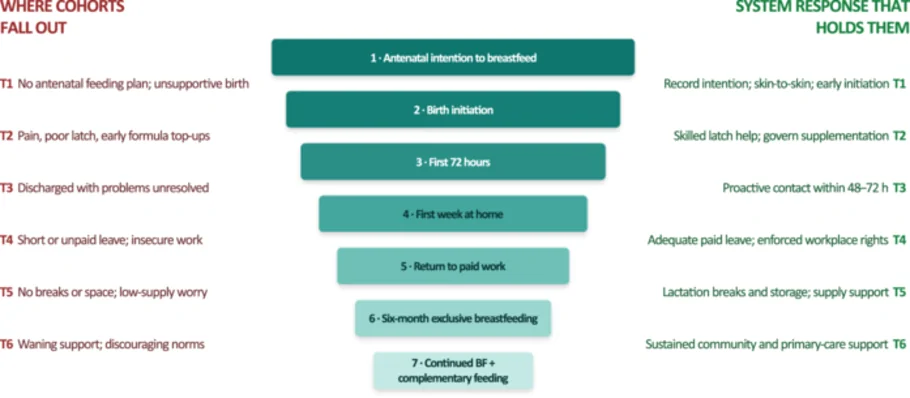
The breastfeeding support cascade. A cohort of women who intend to breastfeed is traced through predictable attrition points, from birth initiation and the first 72 h through the first week at home, the return to paid work, 6‐month exclusivity, and continued breastfeeding with complementary feeding. Each transition carries characteristic barriers and a corresponding system response that could hold the cohort in effective care. Reading the shortfall as a cascade, as in HIV and chronic‐disease care, locates the points where support should be placed before problems become reasons to stop.

### 3.3 Measuring Intention Without Misrepresenting It

An indicator built on intention must confront the fact that intentions are not static. A woman's feeding goals may shift after birth for reasons that have nothing to do with the quality of her care, including how the birth unfolded, her physical recovery, her infant's temperament, and the circumstances awaiting her at home. Treating an antenatal statement as a fixed benchmark against which later behaviour is judged would misrepresent both the woman and the service caring for her.

Three conventions would make the indicator defensible. Intention should be recorded antenatally and revisited at least twice afterwards, in the first postnatal week and around the return to paid work, because these are the junctures at which support most often fails. A revised goal should be logged as a revision rather than quietly replacing the original, so that analyses can separate goals that changed because a woman's preferences changed from goals abandoned because help did not arrive; the reason for a change is as informative as the change itself. The primary indicator should then count only cessation that a woman herself attributes, at the time, to unresolved problems or absent support, with preference‐driven revision reported separately rather than folded into the same number.

A deeper difficulty is that intentions are partly endogenous to the systems being assessed. Women who expect little help may state modest goals, so a service caring for such a population could appear to perform well simply because expectations were low (Hausman 2012). Intention levels should therefore be reported alongside the gap, and an unusually low intention profile should be read as a signal about the environment rather than as a favourable result.

### 3.4 Equity as the Organising Test

The value of this reframing becomes particularly clear when viewed through an equity lens. The mothers most likely to face barriers are usually those with the least power to negotiate around them, including women in casual and shift‐based work (Dinour and Szaro 2017), migrant women (Izumi et al. 2024), young mothers (Sipsma et al. 2013), sole parents (Rollins et al. 2016), women with limited health literacy (Berkman et al. 2011), women facing racism (Hamner et al. 2021; Jones et al. 2015), and those living far from specialist services (Pronovost 2003; Sinha et al. 2015). Universal promotion campaigns can widen the very gap they aim to close, because better‐resourced families are more able to act on advice, arrange support, and absorb the costs of persistence (Rollins et al. 2016; Victora et al. 2016). A systems approach points instead towards proportionate universalism (Marmot and Bell 2012), a baseline of support for everyone, intensified for those facing the steepest barriers, through proactive home visiting (Yonemoto et al. 2021), interpreters and culturally responsive care (Izumi et al. 2024), peer counsellors (Shakya et al. 2017), culturally safe services (Jones et al. 2015), and targeted enforcement of workplace rights in low‐wage sectors (Dinour and Szaro 2017). Disaggregated indicators are what make this possible, because an equity strategy that cannot see its own gradients cannot be held to account for closing them (Marmot and Bell 2012; Rollins et al. 2016).

### 3.5 The Developmental Implications

Associations between breastfeeding and later developmental outcomes remain important, but they are not central to the present argument. Whether benefits arise from breastfeeding itself, the support systems that enable it, or both, strengthening those support systems remains a defensible policy goal (Horta et al. 2015; Pan et al. 2025; Victora et al. 2015).

The more immediate reason to focus on support is timing. The first postnatal week is when many breastfeeding difficulties emerge or intensify (Brown et al. 2014; Gianni et al. 2019) and when much unwanted cessation is set in motion (Odom et al. 2013). Yet it remains one of the least reliably supported periods of the care pathway (McFadden et al. 2019). If early breastfeeding success depends heavily on what happens during this brief window, then its quality should be measured, managed, and improved rather than assumed. A system that takes early life seriously cannot treat the first postnatal week as a service gap between birth and routine follow‐up.

### 3.6 Guardrails

Such a framework requires clear safeguards. Four matter most.

The first is autonomy. Reclassifying unwanted cessation as a preventable harm must never license pressure on women who choose formula, nor imply that a mother who stops has been failed against her will when in fact she has exercised a free preference (UNICEF UK 2023; World Health Organization WHO, and UNICEF 2018). The indicator of interest is the gap against a woman's own goal, defined by her (Odom et al. 2013), which is why intention must be recorded respectfully and revisited rather than assumed. Formula is safe, necessary for many families, and lifesaving in some circumstances (World Health Organization 2023), and a system that supports it well is part of good care (World Health Organization WHO, and UNICEF 2018).

The second is proportion. The clinical‐quality frame should raise the standard of institutions, not manufacture a new register of blame that migrates from mother to clinician. Front‐line staff are frequently the most under‐resourced actors in the chain, expected to deliver skilled lactation care in minutes they do not have (Gavine et al. 2022; Rollins et al. 2016). Holding a system accountable means resourcing it, not scapegoating its workforce (Braithwaite et al. 2015; Reason 2000).

The third concerns measurement itself. Expanding surveillance of infant feeding carries its own risks. Feminist scholarship has argued for some time that breastfeeding promotion can intensify maternal responsibility and moral scrutiny, converting a physiological process into a test of adequate motherhood (Hausman 2012; Wolf and Joan 2011). An indicator that records whether women met their goals could, handled carelessly, add a further layer of judgement at an already vulnerable time and be experienced as one more way of being found wanting. Three design choices reduce that risk. The unit of reporting should be the institution and never the individual, so that facilities and employers carry rates while women carry none. No clinical target should be set for an individual woman's duration, since targets of that kind generate precisely the pressure the frame exists to remove. And the question put to women should concern the support they received rather than the outcome they achieved, because asking whether help arrived when it was needed locates the deficiency where it belongs. If these conditions cannot be met, the harms of measurement may outweigh its value.

The fourth is context. The argument is sharpest in high‐income systems, where near‐universal initiation collapses into early cessation (McAndrew et al. 2012; Victora et al. 2016) and the failure is manifestly one of support rather than survival. In low‐income settings the enabling‐environment agenda retains its original, life‐and‐death form (Victora et al. 2016), and priorities differ accordingly (Pérez‐Escamilla et al. 2023; Rollins et al. 2016). The framework is broadly applicable, but its priorities should be adapted to local contexts. Singapore is instructive precisely because it is well resourced. Two decades of campaigns, hospital reform, and workplace measures still left exclusive breastfeeding at 6 months hard to reach (Lee et al. 2024), a reminder that wealth alone does not manufacture a breastfeeding‐friendly system and that sustained institutional alignment is what does (Pérez‐Escamilla et al. 2012; Pérez‐Escamilla and Hall Moran 2016).

## 4 A Research and Policy Agenda

The main research questions now concern implementation rather than efficacy. Future work should focus on identifying which combinations of policy, workplace, hospital, and post‐discharge interventions sustainably improve breastfeeding outcomes and reduce inequities. Natural experiments, policy evaluations, and implementation studies are likely to be more informative than further observational analyses linking breastfeeding to later outcomes, valuable though causal work remains where confounding can be tackled credibly (Horta et al. 2015). Before widespread adoption, the proposed indicator should be tested as rigorously as any other quality metric: whether facility‐level unwanted‐cessation rates prove reliable, whether they respond to changes in practice, and whether publishing them improves care or merely generates defensive documentation. These are tractable empirical questions that should be addressed before the indicator is adopted at scale.

It is worth resisting the appeal of narrow technical fixes as substitutes for support. Iron deficiency in infancy is a real concern, and trial evidence indicates that daily iron supplementation can improve infant iron status without shortening exclusive breastfeeding under specified conditions, which is reassuring for families who fear that supplementation undermines the feed (Domellöf et al. 2014; Stelle et al. 2025; World Health Organization 2016). But a supplement addresses one biological risk and leaves the support deficit untouched. It cannot create paid leave, correct a poor latch, answer the phone at three in the morning, counter a targeted advertisement, or reconcile the three different feeding plans a mother has been handed by three different clinicians. The same caution applies to maternal pathways that intersect with feeding, such as care after bariatric surgery, where nutritional and lactation support are easily lost between services (Christenson 2025). The lesson is integration, not another isolated intervention bolted onto a fragmented pathway.

On the policy side, the moves follow directly from the measurement. Make lactation support a component of maternity quality and safety accreditation. Fund proactive contact in the first postnatal week. Guarantee skilled help for pain, mastitis, low‐supply concerns, and faltering weight before these become reasons to stop. Protect paid leave and enforce workplace lactation rights, with attention to the low‐wage sectors where they are least honoured. Regulate commercial marketing, including the digital targeting that now reaches parents through social media, parenting clubs, and search advertising at precisely the moments of fatigue and doubt when they are most susceptible (Baker et al. 2023; World Health Organization 1981; World Health Organization, and UNICEF 2022). And, above all, measure the gap between what women intended and what the system let them achieve, because that gap is the clearest available signal of whether the surrounding institutions are doing their job.

## 5 Conclusion

Breastfeeding surveillance already records the gap between what women hope to achieve and what they ultimately achieve. The problem is not measurement. It is accountability. When large numbers of women stop breastfeeding earlier than they intended, that outcome should be interpreted not only as a behavioural result but also as evidence about the systems surrounding them. The intention‐to‐cessation gap is already measured, its risk factors are already known, and its social patterning has already been documented. What remains missing is accountability for the gap. Treating unwanted early cessation as an accountable quality indicator would shift attention from auditing mothers to improving the institutions that shape feeding outcomes. Until then, breastfeeding statistics will continue to describe a problem without assigning responsibility for solving it.

## Author Contribution

The Perspective is sole‐authored ‐ Jimmy Chun Yu Louie wrote the manuscript in its entirety and is the guarantor of the work.

## Funding

The author has nothing to report.

## Conflicts of Interest

The author declares no conflicts of interest.

## Policy on Using ChatGPT and Similar AI Tools

During the preparation of this work the author used Claude sonnet 5.0 (https://claude.ai) in Jul and Sep 2026 in order to improve the grammar and clarity of the text he drafted. After using these tools/services, the corresponding author reviewed and edited the content as needed and takes full responsibility for the content of the publication.

## Data Availability

Data sharing not applicable to this article as no datasets were generated or analysed during the current study. No primary data were generated. All sources are cited.
